# Efficient Production of Hepatocyte-like Cells from Human-induced Pluripotent Stem Cells by Optimizing Growth Factors

**Published:** 2018-05-01

**Authors:** Z. Jafarpour, M. Soleimani, S. Hosseinkhani, M. H. M. H., P. Yaghmaei, N. Mobarra, B. Geramizadeh

**Affiliations:** 1 Department of Biology, Science and Research Branch, Islamic Azad University, Tehran, Iran; 2 Department of Hematology, Faculty of Medical Science, Tarbiat Modares University, Tehran, Iran; 3 Department of Biochemistry, Faculty of Biological Sciences, Tarbiat Modares University, Tehran, Iran; 4 Transplant Research Center, Shiraz University of Medical Sciences, Shiraz, Iran; 5 Metabolic Disorders Research Center, Department of Biochemistry, School of Medicine, Golestan University of Medical Sciences, Gorgan, Iran

**Keywords:** Induced pluripotent stem cells, Hepatocyte-like cells, differentiation; Induced pluripotent stem cells

## Abstract

**Background:**

Generating hepatocytes with complete liver functions is still a challenge and developing more functional hepatocytes is needed.

**Objective:**

To compare various differentiation factors and protocols and introducing a preferable protocol to differentiate human-induced pluripotent stem cells (hiPSCs) into hepatocyte-like cells (HLCs).

**Methods:**

After 3 days of the endoderm differentiation of hiPSCs, the cells were incubated with 5 hepatocyte differentiation culture media, protocols (P), for 14 days—P1: hepatocyte growth factor and fibroblast growth factor-4 (FGF-4) for the first week and oncostatin-M and dexamethasone for the second week; P2: similar to P1 but FGF4 was used in both the first and second weeks; P3: similar to P1 but FGF-4 was not used; P4: similar to P1 but FGF-4 and dexamethasone were not used; and P5: similar to P1 but FGF-4 and oncostatin-M were not used. After 17 days, characterization was done by qRT-PCR, immunofluorescence and ELISA.

**Results:**

The mRNA expression levels of hepatocyte markers (albumin, cytokeratin-18, tyrosine aminotransferase, hepatocyte nuclear factor-4α, cytochrome-P450 7A1) increased significantly (p<0.05) in the differentiated cells by 5 different protocols. Furthermore, significant protein expression and secretion of albumin were detected in the differentiated cells by 5 different protocols. In P3, the differentiated cells had the highest exhibit of hepatocyte characteristics and in P4 they had the lowest. Moreover, in P1 and P2 similar results were observed.

**Conclusion:**

Since P3 gave us the best results among all protocols, we recommend it as an efficient protocol to differentiate the functional HLCs from hiPSCs, which can improve cell therapies.

## INTRODUCTION

Transplantation of the liver is restricted due to the shortage of organ donors. Therefore, the transplantation focus can be shifted to human hepatocytes differentiated from stem cells [1]. Human-induced pluripotent stem cells (hiPSCs) are an appropriate source for producing different types of cells such as hepatocytes in vitro. Different from other stem cells and similar to embryonic stem cells (ESCs), hiPSCs can proliferate unlimitedly, while preserving their potency [2]. In addition, iPSCs have a self-renewing characteristic and a potential to differentiate into three embryonic germ layers [3]. Generating iPCs from somatic cells has increased the possibility of producing patient’s specific cell types of all different lineages [2]. Furthermore, integration of virus-associated genotoxicity is no longer a problem in clinical usage because of producing integration-free iPSCs [4]. 

Differentiated cell types including hepatocytes, generated from iPSCs, have many potential therapeutic usages such as tissue replacement and drug discovery support systems [2, 5]. Various protocols for the differentiation of stem cells into hepatocytes have been reported in previous studies [2, 6-8]. The foundation for developing these protocols for human pluripotent stem cells is the mouse ESCs [6]. These protocols are different from each other in terms of their types of stem cells, the period for each stage of differentiation, used feeders for stem cells, and type and quantity of the used factors or chemical components. The used factors include hepatocyte growth factor (HGF), fibroblast growth factor-4 (FGF-4), oncostatin M (OSM, from interleukin 6 group) and chemical components including dexamethasone (Dex). These factors are signals influencing the differentiation of hepatocytes [9]. HGF, for instance, has a main function in the regeneration and development of the hepatocytes. Moreover, the differentiation of hepatocytes that are not actively proliferating, is induced by HGF [7]. 

Forkhead box A2 (FOXA2), and SRY-box containing gene 17 (SOX17) are definitive endoderm (DE) marker genes. Albumin (ALB), cytokeratin-18 (CK18), tyrosine aminotransferase (TAT), hepatocyte nuclear factor 4α (HNF4α), and cytochrome P450 7A1 (CYP7A1) are hepatocyte marker genes [6].

In our previous study [6], we differentiated hiPSCs into hepatocyte-like cells (HLCs) using hepatocyte differentiation culture media, including HGF, FGF4, FBS, OSM, and Dex on hMSCs feeder. However, none of the past studies have resulted in producing a fully functioning hepatocyte. In order to have an efficient differentiation, the used protocols should be optimized [1]. There are no studies that compare the multiple protocols of producing HLCs from hiPSCs, to the best of our knowledge. 

In this study, we evaluated the effects of various factors, FGF4, OSM, and Dex, on the differentiation of hepatocytes from hiPSCs and compared different hepatocyte differentiation protocols regarding their efficiency, and recommended an efficient protocol to produce functional hepatocytes for clinical applications. 

## MATERIALS AND METHODS

hMSCs Culture

Human adult bone marrow-derived mesanchymal stem cells (MSCs) were acquired from Stem Cells Technology Research Center, Tehran, Iran, under supervision of its ethics committee. These cells were in passage five and were applied as feeder layer. They were cultured in Dulbecco’s modified Eagle’s medium (DMEM, Gibco, USA) supplemented with 15% (v/v) fetal bovine serum (FBS, Gibco, USA). 

When the cells reached 80%–90% confluency, they were inactivated by mitomycin-C (10 µg/mL, Santa Cruz Biotechnology, USA) [10]. 

HiPSCs Culture

HiPSCs obtained from Stem Cells Technology Research Center, Tehran, Iran (passages 8–14) were cultured on a layer of hMSCs in DMEM/F12 supplemented with 10% FBS stem cell qualified replacement, 10% knockout serum replacement, 1 mM L-glutamine (all from Gibco, USA), 1% non-essential amino acids (PAA, Austria), 1% penicillin/streptomycin and 10 ng/mL basic fibroblast growth factor (Peprotech, USA). They were incubated in a standard condition with 95% humidity and 5% CO_2_ at 37 °C.

DE and Hepatocyte Differentiation of hiPSCs

To induce the DE differentiation, hiPSC colonies (without hMSCs) were passaged into gelatin-coated plates and incubated for three days with RPMI-1640 medium (Gibco, USA) supplemented with 100 ng/mL activin A (R&D, USA), 0.5 mg/mL albumin fraction V (Sigma-Aldrich, USA) and insulin-transferrin-selenite (ITS, Gibco, USA). The concentration of ITS was 0% on the first day, 0.1% on the second day, and 1.0% on the third day [11]. For hepatocyte differentiation, the medium was replaced to continue the differentiation for 14 days later using five different protocols (P). These five protocols included P1: DE cells were incubated for seven days with hepatocyte culture medium (HCM, Lonza, Switzerland) supplemented with 20 ng/mL hepatocyte growth factor (HGF, R&D, USA) and 20 ng/mL fibroblast growth factor-4 (FGF-4, R&D, USA). The medium was changed every two days. Then, the medium was replaced and the cells were incubated for seven days later with HCM supplemented with 20 ng/mL oncostatin M (OSM, R&D, USA), 0.1 µM Dex (R&D, USA), 1% non-essential amino acids, and L-glutamine; P2: The same protocol as P1 was used, but FGF-4 was also added to the medium in the second seven days; P3: The same protocol as P1 was used, but FGF-4 was totally omitted from the protocol; P4: The same protocol as P1 was used, but FGF-4 and Dex were omitted; P5: The same protocol as P1 was used, but FGF-4 and OSM were omitted (Table 1).

**Table 1:** Used main factors in the five studied protocols. At hepatocyte differentiation stage, the definitive endoderm cells were incubated with five different culture media for 14 days. P1: Protocol 1, P2: Protocol 2, P3: Protocol 3, P4: Protocol 4, and P5: Protocol 5

Protocols	Used main factors at the first week	Used main factors at the second week
P1	HGF, FGF4	OSM, Dex
P2	HGF, FGF4	FGF4, OSM, Dex
P3	HGF	OSM, Dex
P4	HGF	OSM
P5	HGF	Dex

Immunofluorescent Staining

The produced cells were washed with PBS, fixed in 4% paraformaldehyde (Sigma, USA) at 4 °C for 20 min and then at room temperature (RT) for 5 min. After washing with PBS, they were incubated with 0.2% Triton X-100. The cells were washed with PBS, blocked with 10% goat serum (Gibco, USA) at RT for 30 min and then removed. They were incubated at 4 °C overnight with a primary antibody; mouse monoclonal anti-albumin (1:200, R&D, USA). After washing with PBS, the cells were incubated at RT for 1 hr with a PE conjugated secondary antibody, goat anti-mouse (1:100, R&D, USA). Then, they were washed with PBS. The nuclei were stained with DAPI (1 µg/mL) (Sigma, USA). The cells were analyzed using a fluorescent microscope (Nikon, Japan). 

Quantitative Real-time PCR (qRT-PCR)

Total RNA was extracted from the cells using Trizol reagent (Gibco, USA). In the reverse transcription reaction, 2 µg of total RNA was used with the cDNA synthesis kit (Fermentas, USA) and random hexamer as primers, according to the manufacturer’s instructions. qRT-PCR was carried out using SYBR Premix ExTaq Master (Takara, Japan) and monitored using a Rotor Gene 6000 system (Corbett, Australia). Data were normalized to an endogenous control gene (Beta2M), calibrated to the hiPSCs, and analyzed by the comparative threshold cycle method. The primers are listed in Table 2. They were obtained from Stem Cells Technology Research Center (Tehran, Iran). The PCR reaction was carried out at 95 °C for 2 min, followed by 40 cycles of 95 °C for 5 s and 60 °C for 45 s. The reaction was made of 6.5 µL of SYBR Green PCR Master Mix, 1 µL of template cDNA, 0.5 µL 10 µM of reverse primer, 0.5 µL 10 µM of forward primer, and 4.5 µL of water [11]. 

**Table 2:** Primers used in qRT-PCR analysis

Gene	Sequences, (forward and reverse)	T_m _(°C)	Product size (bp)
Beta2M	5’-ATG CCT GCC GTG TGA AC-3’ 5’-ATC TTC AAA CCT CCA TGA TG-3’	56	91
TAT	5’-CTT CCT CAA GTC CAA TGC TG-3’ 5’-TGT TCC ATC TCA ATT CCA ACC-3’	58	113
CK18	5’-TGG CGA GGA CTT TAA TCT TGG-3’ 5’-CTC AGA ACT TTG GTG TCA TTG G-3’	55	128
ALB	5’- GAGACCAGAGGTTGATGTGATG-3’ 5’-AGGCAGGCAGCTTTATCAGCA-3’	58	186
CYP7A1	5’-GGA ATT AGG AGA AGG CAA ACG-3’ 5’-CAC CAA ATT GCA GAG CAC AG-3’	60	82
HNF4α	5’-CTTCTTTGACCCAGATGCCAAG-3’ 5’-GAGTCATACTGGCGGTCGTTG-3’	58	78

Albumin Secretion 

After 17 days, the secreted ALB was measured in cell culture media using the human ALB ELISA kit (Bethyl, USA) according to the manufacturer’s instructions. Anti-human ALB antibody had been pre-adsorbed on the surface of microtiter wells. The cell culture media were added to each well, incubated at RT for 60 min and then the plate was washed with TBS buffer. Next, anti-ALB detection antibody was added, incubated at RT for 60 min and then washed. Then, a strepavidin-conjugated horseradish peroxidase (HRP solution) was added, incubated at RT for 30 min and then washed. The TMB substrate solution was then added, incubated in the dark at RT for 30 min. Finally, H_2_SO_4_ was added to stop the reaction. Absorbance was measured at 450 nm on a plate reader. The ALB concentration in cell culture media was calculated using a standard curve.

Statistical Analysis

All experiments were performed in triplicate. The data were expressed as a mean±SD. Means of three or more groups were compared using one-way ANOVA. A p value <0.05 was considered statistically significant.

## RESULTS

Evaluation of Differentiated Cells and Phenotype Characteristics of Cells

**Figure 1:**
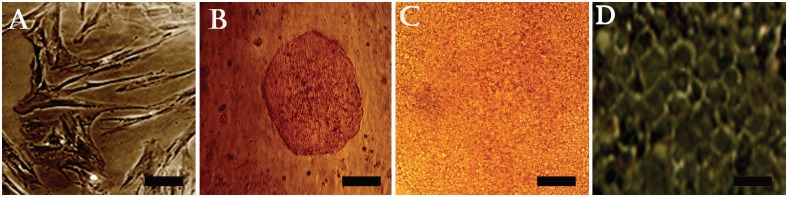
Phase contrast microscopy images of A) hMSCs (scale bar=50 μm); B) hiPSC colonies on hMSCs feeder (scale bar=200 μm); C) differentiated hiPSCs on the third day (scale bar=800 μm); and D) differentiated hiPSCs on the 17th day (scale bar=100 μm)

The morphology of hMSCs, which were being used as a feeder, was fibroblastic (Fig 1A). hiPSC colonies, which were cultured on hMSCs, were circular and tightly packed and had big nuclei and little cytoplasm (Fig 1B). In order to induce DE differentiation, the hiPSCs (without feeder layer) were transferred into cell culture plates and differentiated into DE cells in three days (Fig 1C). Then, they were differentiated into HLCs in 14 days using five different protocols (Table 1). After 17 days, the produced cells were evaluated based on their morphology. The produced cells of all five protocols changed into cubical shapes, which is a typical characteristic of hepatocytes (Fig 1D).

The Expression of DE and Hepatocyte Genes

**Figure 2:**
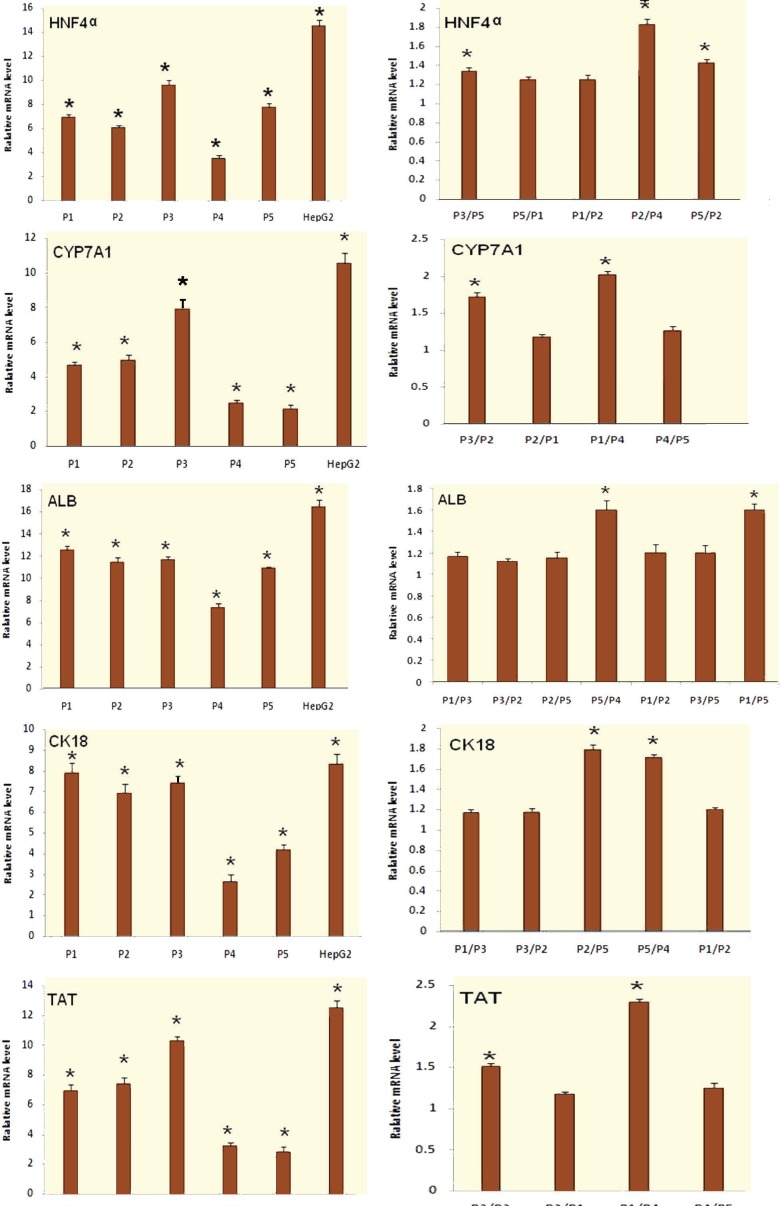
qRT-PCR analysis of hepatocyte markers. Beta2M was evaluated as the housekeeping gene. Values are mean±SD; * p<0.05; P1: Protocol 1, P2: Protocol 2, P3: Protocol 3, P4: Protocol 4, and P5: Protocol 5

The expression levels of DE differentiation markers (SOX17, FOXA2) were studied in differentiated hiPSCs in comparison with the undifferentiated hiPSCs on the third day using qRT-PCR. The expression levels of both genes increased significantly (p<0.05) in the differentiated hiPSCs using the five protocols. The expression level of FOXA2 increased by a mean±SD of 37.43±2.04 times in the differentiated hiPSCs in comparison with the undifferentiated hiPSCs. The mean±SD expression level of SOX17 increased by 35.02±2.11 times in the differentiated hiPSCs in comparison with the undifferentiated hiPSCs. After 17 days, the expression levels of hepatocyte differentiation markers (ALB, CK18, TAT, HNF4α, CYP7A1) were evaluated using qRT-PCR in the differentiated hiPSCs in comparison with the undifferentiated hiPSCs. The expression levels of all these hepatocyte genes increased significantly (p<0.05) in the differentiated cells using all five protocols. For example, the mean±SD expression level of CYP7A1 increased by 4.65±0.23 times in the differentiated hiPSCs using P1, in comparison with the undifferentiated hiPSCs, by 4.97±0.31 using P2, by 7.89±0.52 using P3, by 2.47±0.2 using P4, by 2.14±0.24 using P5, and by 10.53±0.61 in HepG2 cells (the positive control) (Fig 2). The expression levels of other hepatocyte markers (ALB, CK18, TAT, HNF4α) in the differentiated hiPSCs in comparison with the undifferentiated hiPSCs are shown in Figure 2.

The expression levels of TAT and CYP7A1 in differentiated cells using P3 were the highest among all five protocols examined, while P4 and P5 resulted in the lowest levels. The expression levels of these genes, among the following protocols were significantly different (p<0.05 for P3/P2, P3/P1, P1/P4, P1/P5, P2/P4 and P2/P5). The expression levels of these genes in differentiated cells using P2 were similar to P1, and using P4 was similar to P5. Therefore, the differences observed among the protocols were not significant (p>0.05 for P4/P5 and P2/P1).

The expression levels of CK18 in differentiated cells using P1, P2 and P3 were the highest among all five protocols, while P4 showed the lowest results. The expression levels of this gene, between the following protocols were significantly different (p<0.001 for P1/P5, P3/P5, P2/P5 and P5/P4). The expression levels of this gene in differentiated cells using P1, P2 and P3 were similar. Therefore, the differences observed among the protocols were not significant (p>0.05 for P1/P3, P1/P2 and P3/P2).

The expression level of HNF4α in differentiated cells using P3 was the highest among all five protocols while P4 showed the lowest results. The expression levels of this gene among the following protocols were significantly different (p<0.05 for P3/P5, P3/P1, P3/P2, P5/P2, P5/P4 and P2/P4).The expression levels of this gene in differentiated cells using P1 and P2 were similar. Therefore, the differences were not significant (p>0.05 for P1/P2). 

The expression levels of ALB in differentiated cells using P1, P2 and P3 were the highest among all five protocols while P4 showed the lowest results. The expression levels of this gene, among the following protocols were significantly different (p<0.05 for P1/P4, P3/P4, P2/P4, P5/P4 and P1/P5). The expression levels of this gene in differentiated cells using P1, P2 and P3 were similar. Therefore, the differences were not significant (p>0.05 for P1/P3, P1/P2 and P3/P2) (Fig 2).

Immunofluorescence Analysis of ALB

After 17 days, protein expression level of ALB (the percentage of ALB positive cells) in produced cells was evaluated. This analysis demonstrated that after 17 days a significant expression level of ALB was detected in generated cells by five different protocols. The mean±SD protein expression level of ALB in the produced cells by P3 (38.24%±6.395) was at the highest level; in produced cells by P4 (15.38%±3.069), it was at the lowest level in comparison with the produced cells by other protocols. This protein was not detected in undifferentiated hiPSCs.

**Figure 3:**
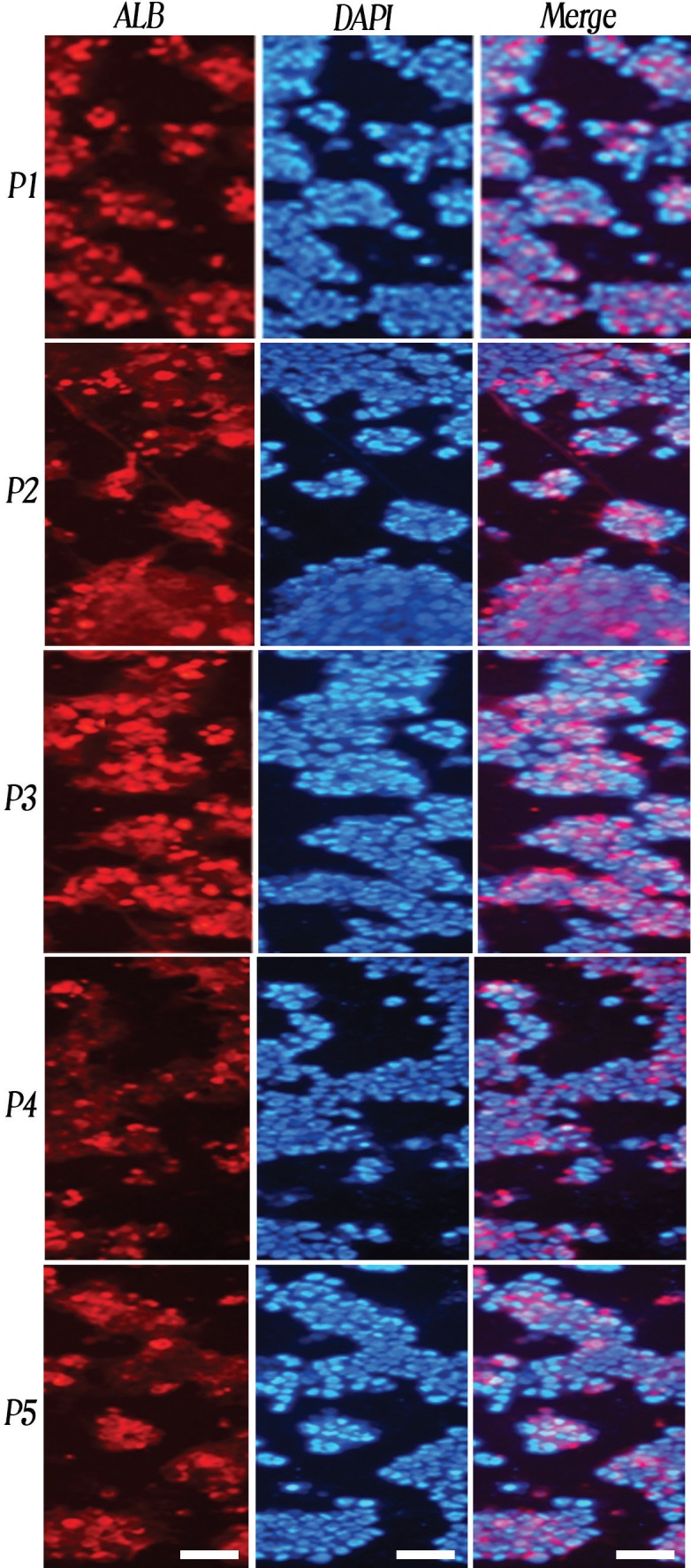
Immunofluorescent staining of expression of hepatocyte marker ALB in differentiated hiPSCs using different protocols. Nuclei were stained with DAPI. Scale bar=100 μm; P1: Protocol 1, P2: Protocol 2, P3: Protocol 3, P4: Protocol 4, and P5: Protocol 5

**Figure 4:**
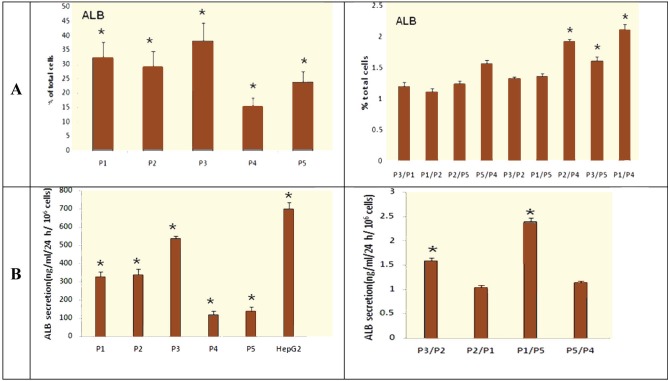
The analysis of hepatocyte markers. A) The percentage of the cells expressing ALB on the 17^th^ day of the differentiation using immunofluorescent analysis; B) ALB secretion of produced cells on the 17^th^ day of the differentiation. Values are mean±SD of three independent experiments. *p<0.05; P1: Protocol 1, P2: Protocol 2, P3: Protocol 3, P4: Protocol 4, and P5: Protocol 5

Significant differences were observed in the produced cells using the following protocols (p<0.05 for P3/P4, P2/P4, P3/P5 and P1/P4); however, no significant differences were detected in the produced cells using the following protocols (p>0.05 for P2/P1 and P5/P4) (Figs 3, 4A). 

Secretion Analysis of ALB

We carried out this analysis to check the ability of one of the hepatocyte functions in the generated cells. The results showed that after 17 days, the level of produced ALB increased significantly (p<0.05) in differentiated cells using five different protocols, in comparison with undifferentiated hiPSCs. The undifferentiated hiPSCs did not produce ALB. The mean±SD level of ALB secretion in differentiated iPSCs using P3 (537.24±14.66 ng/mL) was at the highest level while in differentiated iPSCs using P4 (119.63±17.38 ng/mL), it was at the lowest level in comparison with the differentiated cells using other protocols. Significant differences were found in the produced cells using the following protocols (p<0.05 for P3/P2, P3/P5 and P1/P5), but no significant differences were observed in the produced cells using following protocols (p>0.05 for P2/P1 and P5/P4) (Fig 4B).

## DISCUSSION

Generating hepatocytes with a quality of complete liver functions is still an unsolved problem in medical sciences, even though the quantity of produced hepatocytes for clinical usage is sufficient. In fact, the function of the currently produced hepatocytes is not as efficient as natural hepatocytes. Therefore, it is necessary to clarify and use major developmental factors. More efforts are required to set up better protocols to differentiate the stem cells into mature hepatocytes for cell therapies [1]. To achieve this goal, we studied different protocols to differentiate the stem cells into hepatocytes [12-15]. Activin-A is a very important factor in the DE differentiation stage and a high concentration of this factor can induce DE differentiation [16]. Therefore, we used activin-A in the DE differentiation stage. Moreover, we realized that HGF is being used in most of the hepatocyte differentiation protocols; it is the most efficient factor in the hepatocyte differentiation stage. Therefore, we used HGF in the hepatocyte differentiation stage of all of our protocols. We evaluated the effects of other factors, namely FGF4, Dex and OSM to set up the best protocol. At the first stage, hiPSCs (without hMSCs) were transferred into gelatin-coated plates and differentiated into DE cells during three days using the preferable protocol, which was introduced in our previous report [11], combination of activin-A, ITS and ALB fraction-V (Fig 1). At the hepatocyte differentiation stage, the produced DE cells were differentiated into HLCs using five different protocols. In fact, in order to compare the effects of the presence or absence of the factor(s), we eliminated one or two factors in each protocol. In P1, we used all the factors. During the first week, HGF and FGF4, and during the second week, Dex and OSM were used. In P2, FGF4 was used in both the first and second weeks. In P3, FGF-4 was not used and completely removed from the protocol. Therefore, we compared the effect of using FGF4 among P1, P2 and P3. In P4, neither FGF4 nor Dex was used. As a result, the effect of using Dex was compared between P3 and P4. Finally, in P5, FGF4 and OSM were not used. Consequently, we studied the effect of using OSM between P3 and P5. We also drew other useful information by comparing the produced cells using other protocols. 

Studies demonstrate that the hepatocytes, which are differentiated from the stem cells, exhibit metabolic and biochemical characteristics similar to mature hepatocytes [17]. Therefore, we can assess the efficiency of the differentiation by evaluating these characteristics. In our study after 17 days, qRT-PCR results showed that the mRNA expression levels of all the analyzed hepatocyte markers increased significantly (p<0.05) in the differentiated cells using five different protocols (Fig 2). Immunofluorescence analysis showed that significant expression levels of ALB were detected the differentiated cells using the five protocols and 15.38% (in P4) to 38.24% (in P3) of the produced cells expressed ALB (Figs 3, 4A). ELISA demonstrated that the levels of produced ALB were significant in the differentiated cells using the five protocols, and were 537.24 (in P3) to 119.63 ng/mL (in P4) (Fig 4B). This information was consistent with the previous studies in regards to the hepatocyte differentiation of the stem cells [18-21]. In our study, the qRT-PCR, immunofluorescence and ELISA results indicated that hiPSCs were differentiated into HLCs using all five protocols and exhibited the evaluated hepatocyte characterizations. However, the degree of differentiation was not the same in the differentiated cells of the five protocols used. Among all produced cells, the differentiated cells using P3 had the highest mRNA expression level of TAT, CYP7A1 and HNF4α, and also ALB secretion. The mRNA expression levels of CK18 and ALB genes and also the protein expression level of ALB in the differentiated cells by P1, P2 and P3 were similar to each other and these expression levels were at the highest level in comparison with the other differentiated cells. Therefore, P3 had the best results compared to other protocols such as P1 and P2. It seems that the omission of FGF4 in P3 caused better results in comparison with the use of FGF4 in P1 and P2. These results were compatible with other report [5] that showed the use of FGF4 decreases the effects of HGF and activin-A on hepatocyte differentiation. In the mentioned report, the effects of growth factors such as HGF, FGF4 and activin-A were evaluated on differentiation of human ESCs into early hepatocytes. The highest differentiation was observed in the protocol differentiated, first by activin-A and then by HGF [5]. The possible reason of the achieved results would be that cell programs can remarkably alter the cellular responses to the same signals at various stages of development. For instance, in cells which are the origin of the hepatic lineage, FGFs are needed to begin gastrulation and produce the primary anterior endoderm. A transient FGF signal is only required to assist pattern the foregut endoderm. In late gastrula, FGF4 inhibits anterior fate and should be omitted to preserve the foregut progenitors [22, 23]. The results also showed that the mRNA expression levels of CK18, ALB and HNF4α in the differentiated cells using P4 were at the lowest level among other protocols. The mRNA expression levels of TAT and CYP7A1, the protein expression level of ALB and also ALB secretion level in P4 were similar to P5 and they were at the lowest level among other protocols. Therefore, it seems that the elimination of Dex in P4 caused the lowest results in comparison with the other protocols. In agreement with these results, another report [24] demonstrated that for hepatocyte differentiation, Dex induces necessary transcription factors. In the mentioned report, the effects of HGF, epidermal growth factor (EGF) and Dex were evaluated during tissue formation in hepatic organoid cultures from the rat liver. The study also showed that Dex restrains the growth of hepatocytes by suppressing many molecules related to the growth of liver. Dex also improves the differentiation of hepatocyte by influencing expression of many signaling molecules and pathways of signal transduction [24].

The use of OSM in P3 caused better results in comparison with the omission of OSM in P5. This result was consistent with an existing study [25] showing that the stimulation of fetal hepatocytes by OSM increases the expression of different types of genes including HNF4α. That study used fetal hepatocyte derived from mouse embryonic liver, to evaluate the regulation of xenobiotic responses at fetal liver development. According to these results, P3 was found as the best protocol to differentiate hiPSCs into HLCs among the used protocols in the present study. Similar to other studies [26-29], our results demonstrated that HGF could induce hepatocyte differentiation in different media.

In the hepatocyte differentiation in another previous study [8], FGF4 was used in both the first and second weeks, similar to P2. However, we showed that the results of P1 and P2 were similar and increasing the period of using FGF4 did not have any effect on the results. 

In our previous report [6], the hepatocyte differentiation medium of hiPSCs included HGF, FGF4, OSM, Dex and FBS. However, in the current study, we did not use FBS because it was an unclear supplement and could transfer contaminations to the culture media [11]. Moreover, we did not use FGF4 in P3 introduced as the preferable protocol of this study. Therefore, the difference between P3 with previously published protocol [6] was lack of FGF4 and FBS.

In another study [30], similar to P5, the hepatocyte differentiation medium of hiPSCs included HGF and Dex. In both studies, FGF4 and OSM were not used in the culture medium. However, P3, in which we used OSM, exhibited better results in comparison to P5.

Altogether, in the present study, we compared different differentiation factors and protocols and introduced an efficient protocol to differentiate hiPSCs into HLCs. For better understanding of the reasons for the observed differences, further research studies should be done on the cellular signaling pathways of differentiating human stem cells into hepatocytes. More studies on *in vivo* function of the produced HLCs can provide more possibility to use them in cell transplantation therapies.
